# Hearing Smarter: A Scoping Review of Artificial Intelligence Implementation in Hearing Devices

**DOI:** 10.3390/jcm15208124

**Published:** 2026-10-20

**Authors:** Cameron Hanegan, Aya Andos, Rahul Ranganath, Ashley Ezeldin, Hunter Price, Jordan Ford, Andrew Mangan, Gresham Richter

**Affiliations:** 1 Department of Otolaryngology-Head and Neck Surgery, University of Arkansas for Medical Sciences, Little Rock, AR 72205, USA; chanegan@uams.edu (C.H.); aandos@uams.edu (A.A.); amangan@uams.edu (A.M.); 2 College of Medicine, University of Central Florida, Orlando, FL 32827, USA; 3 School of Osteopathic Medicine, A.T. Still University in Arizona, Mesa, AZ 85206, USA; 4 College of Medicine, University of Arkansas for Medical Sciences, Little Rock, AR 72205, USA; rranganath@uams.edu (R.R.); amezeldin@uams.edu (A.E.); hrprice@uams.edu (H.P.); jford4@uams.edu (J.F.); 5 Department of Pediatric Otolaryngology, Arkansas Children’s Hospital, Little Rock, AR 72202, USA

**Keywords:** hearing loss, hearing aid technology, artificial intelligence, machine learning, deep learning, signal processing, speech enhancement, noise reduction

## Abstract

**Background/Objectives**: Hearing loss affects over 1.57 billion people worldwide, and many who could benefit from hearing aids do not wear them. Poor speech understanding in noise is one of the main complaints, and several artificial intelligence (AI) models have been proposed to address it. This review aims to characterize AI applications in hearing devices and offers a framework for advancing from model development to clinical application. **Methods**: Following PRISMA-ScR guidelines, PubMed/MEDLINE, Embase, CINAHL, Web of Science, and APA PsycInfo were searched for peer-reviewed original studies published from 2010 to 2025. Data regarding AI task, architecture, input, training method, validation, and latency were collected for each model. **Results**: In total, 46 studies met the inclusion criteria. Speech enhancement was the most common task (58.7%), and deep neural networks were the most common architecture (34.8%). Training was predominantly offline (93.5%) and supervised (91.3%). Validation relied mainly on database/simulation (71.7%) and normal-hearing listeners (39.1%); only 1 model (2.2%) was validated using hearing-impaired listeners in real-world conditions. Ten models (21.7%) met the latency requirements for hearing aid use. **Conclusions**: AI has been applied across many hearing device tasks, but most models remain early in development. Clinical translation will require on-device implementation and real-world validation in hearing-impaired listeners.

## 1. Introduction

Hearing loss is among the most prevalent chronic health conditions worldwide. Over 1.57 billion people are estimated to be living with hearing loss, making it the third-leading cause of years lived with disability; this number is expected to increase to about 2.45 billion people by 2050 [1]. Hearing loss is associated with long-term health consequences, one of the most significant being dementia. A recent meta-analysis of 50 studies reported a 16% increase in dementia risk for every 10-decibel (dB) hearing loss [2]. In addition to cognitive decline, hearing loss is associated with social isolation, depression, reduced quality of life, and increased mortality [3]. Hearing aids are a primary treatment for hearing loss; however, their benefits depend heavily on consistent use. Some studies report that approximately 70 to 80% of adults who could benefit from hearing aids do not use them [3,4,5]. A major complaint with current hearing aids is that patients have difficulty understanding speech in noisy environments [3,6].

This speech-in-noise problem is also the central limitation on signal processing with conventional hearing devices. Conventional single-microphone, non-spatial noise reduction algorithms have historically failed to improve speech intelligibility; these algorithms provide gains in listening comfort and listening effort, but they do not increase the number of understood words [3]. Multi-microphone spatial or beamforming processors have subsequently been implemented in hearing aids in an effort to overcome this limitation [7]. Most commercially available hearing aids today reduce noise using spatial selectivity, such as directional microphones and beamforming, which improves intelligibility only when the target and noise are spatially separated. Traditional amplification methods further propagate the speech-in-noise limitation by relying on fixed, rule-based fittings that are not individualized to the real-time acoustic environment [8,9]. Artificial intelligence (AI) models, including traditional machine-learning (ML) and modern deep-learning approaches, have been proposed to address the persistent challenge of speech processing in complex acoustic environments.

AI methodologies proposed for hearing devices span a broad range of computational approaches. These approaches range from conventional machine learning (ML) and Bayesian frameworks to deep-learning architectures, which include deep neural networks (DNNs), convolutional neural networks (CNNs), recurrent neural networks (RNNs), attentive recurrent networks (ARNs), generative adversarial networks (GANs), and encoder–decoder architectures such as U-Net and Wave-U-Net. Researchers have also developed specialized hybrid architectures, including Deep Computational Auditory Scene Analysis (Deep CASA), Deep Complex Convolutional Recurrent Networks (DCCRNs), and Gated Convolutional Recurrent Networks (GCRNs), which combine multiple neural-network components to accomplish their tasks. The growing diversity of AI architectures reflects the increased efforts to overcome the limitations of traditional signal processing techniques and improve hearing device performance under real-world acoustic conditions.

Over the past decade, deep-learning methods have rapidly advanced acoustic signal processing for hearing devices. Deep-learning approaches, including DNNs, CNNs, RNNs, and related architectures, have significantly improved speech intelligibility and noise reduction for hearing devices [3,10,11]. Beyond denoising, AI has been applied to a rapidly expanding set of listening-related tasks, including speech enhancement, wind-noise suppression, acoustic-scene classification, alert-signal detection, speech segregation, source localization, feedback and occlusion management, audio–visual synthesis, adaptive dynamic range optimization, individualized auditory processing enhancement, and auditory attention decoding. The wide variety of applications suggests that AI could reshape how hearing devices process sound across nearly every stage of the acoustic pipeline.

Although AI applications are promising, a gap remains between algorithmic development and clinical translation. Current reviews of AI in audiology and otology focus on hearing screening, diagnosis, and device fitting, and several predate the recent surge in deep-learning methods [12,13,14]. Despite the rapid expansion of AI applications in hearing devices, the literature remains fragmented across different device types, acoustic-processing tasks, and model architectures. No comprehensive synthesis exists on how AI has been applied to hearing devices, which AI approaches have been investigated, and how AI models have been evaluated or implemented in current hearing devices. Accordingly, we conducted a scoping review to identify, characterize, and summarize AI applications in hearing devices; describe the AI architectures and learning approaches used; and examine how researchers have evaluated model performance across different acoustic tasks and validation settings. This review aims to provide a framework for model progression from development to clinical implementation.

## 2. Materials and Methods

We conducted a scoping review of the literature on AI applications in hearing devices following the PRISMA Extension for Scoping Reviews guidelines [15] (Supplementary Table S1). The study protocol was not registered. Our search strategy was developed in collaboration with a research librarian; the final search strategy is provided in Supplementary Table S2. The search was conducted on 26 August 2025, across PubMed/MEDLINE, Embase, CINAHL, Web of Science, and APA PsycInfo databases. The literature search included all publications from January 2010 to August 2025. Search terms were chosen to capture all studies that developed or evaluated AI or ML models intended for use primarily in hearing devices.

Inclusion criteria included the development or evaluation of an AI model intended for use in or in conjunction with hearing aids. All AI model architectures were included in the analysis. Studies involving AI that did not clearly state or demonstrate application to these hearing devices were excluded. Only peer-reviewed, original research articles published in English were considered. The reference lists of included studies and relevant literature reviews were screened to identify additional eligible studies. Studies using AI models exclusively in cochlear implants were excluded.

Studies identified during the literature search were uploaded to Covidence (Veritas Health Innovation, Melbourne, Australia, available at www.covidence.org, last accessed on 10 September 2026), a web-based platform that helps facilitate citation screening, study selection, and data extraction between reviewers. The studies underwent de-duplication and title-and-abstract screening. Full-text articles were then reviewed against the inclusion and exclusion criteria before proceeding to data collection. At least two reviewers screened each study during title-and-abstract screening and full-text screening. A third reviewer resolved any differences between reviewers.

Studies that passed the full-text screening were included in the review, and data were extracted in Covidence using a standardized data-charting form developed by the authors. At least two reviewers independently reviewed each study during data collection. Reviewers resolved discrepancies through discussion and consensus.

The standardized data-charting form captured the following variables: author, year of publication, study design, primary AI task, AI model architecture, model input, reported or derived algorithmic latency, sample size, validation method, and acoustic environment (see Supplementary Table S3). The primary AI task was categorized by the principal hearing-related function that the algorithm performed. AI model architecture was classified based on the computational framework or algorithmic structure underlying the system. Model input referred to the primary data source processed by the model to perform its intended task. Algorithmic latency was defined as the time required for the AI model to process input data and generate an output during signal processing, excluding any hardware-related delays that contribute to total end-to-end latency. The validation method was categorized according to the approach used to evaluate model performance on data not used during training. The acoustic environment was categorized based on the listening conditions evaluated in each study.

Objective outcomes, subjective outcomes, and future directions were described narratively. Objective outcomes included quantifiable measures of model performance, whereas subjective outcomes included participant-reported assessment of the model’s perceived effectiveness.

Algorithmic latency was used to assess whether the AI model was potentially feasible within hearing aid latency requirements. Each model was classified against the latency budget of 5 to 10 milliseconds (ms), representing the upper limit of processing delay before self-voice coloration, echo, and sound quality degradation become perceptible [16]. Feasibility was categorized as “Yes,” “Partial/Conditional,” “No,” “Unable to Determine,” or “Not Applicable.” A model was categorized as “Partial/Conditional” when the model could meet the latency requirement only under specific stated conditions.

The data were summarized, and all figures and tables were created using Microsoft Excel for Microsoft 365 (version 2806; Microsoft Corporation, Redmond, WA, USA).

## 3. Results

**Figure 1:**
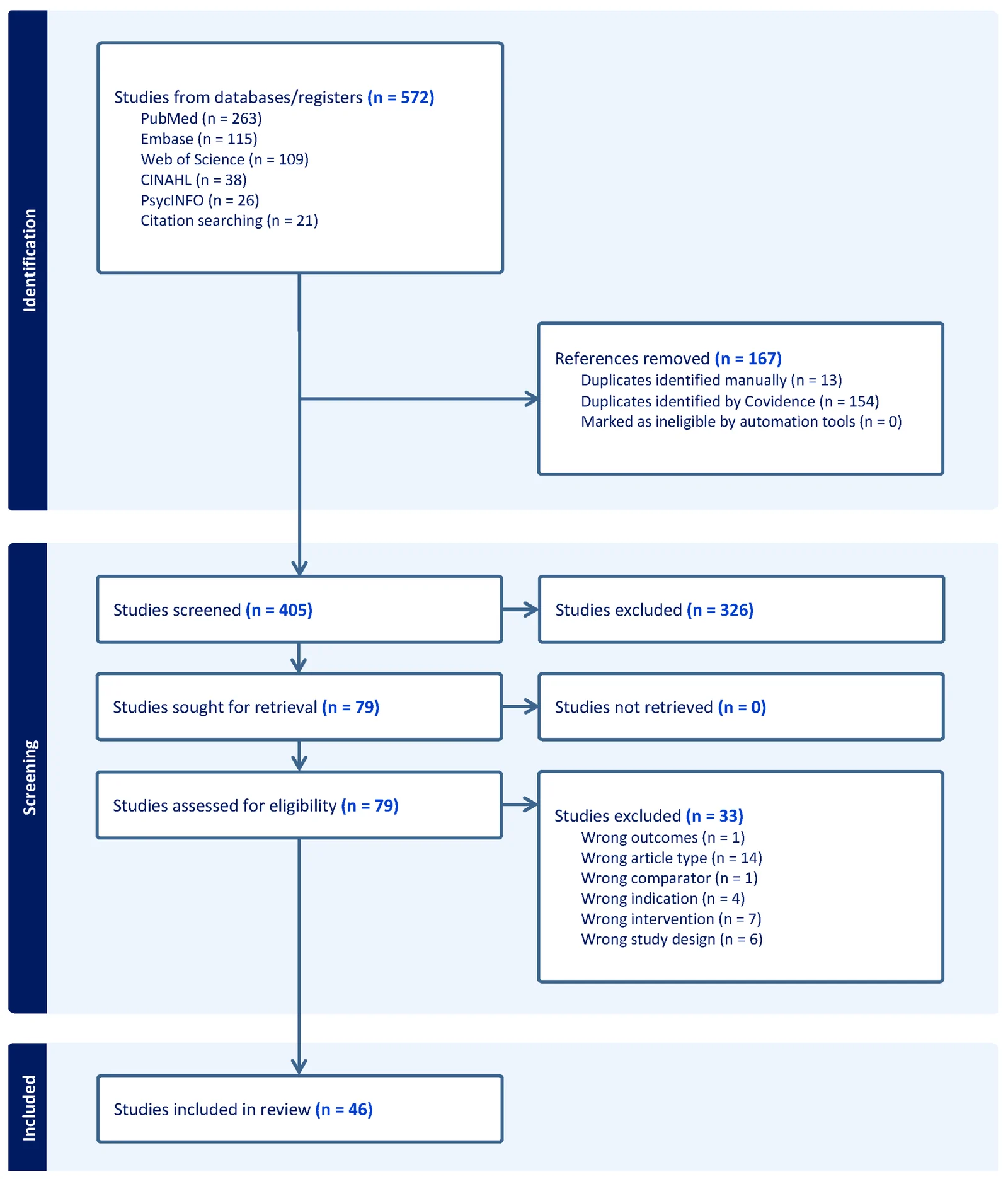
PRISMA-ScR flow diagram. The literature search identified 572 studies. These studies underwent de-duplication, title-and-abstract screening, full-text screening, and final inclusion.

In total, 572 studies were identified through database and citation searches. After removing duplicates, 405 studies underwent title-and-abstract screening, and 79 full-text studies were subsequently assessed for eligibility. Of these, 46 studies met the inclusion criteria and were included in the review. The PRISMA-ScR flow diagram (Figure 1) summarizes the study selection process. Table 1 presents descriptive characteristics of the studies, and Supplementary Table S3 provides the full per-study extraction. Several variables were not mutually exclusive, and a single study could report multiple learning methods, acoustic testing environments, validation methods, and training or testing datasets. As a result, counts for those variables may sum to more than 46. All percentages are reported relative to the total of 46 publications unless otherwise stated.

The studies included a mix of engineering (*n* = 25, 54.3%) and translational (*n* = 21, 45.7%) research, with most involving AI model development and validation (*n* = 41, 89.1%). A small number of included publications included laboratory perceptual evaluation (*n* = 4, 8.7%) and a single real-world effectiveness study using ecological momentary assessment (*n* = 1, 2.2%).

**Figure 2:**
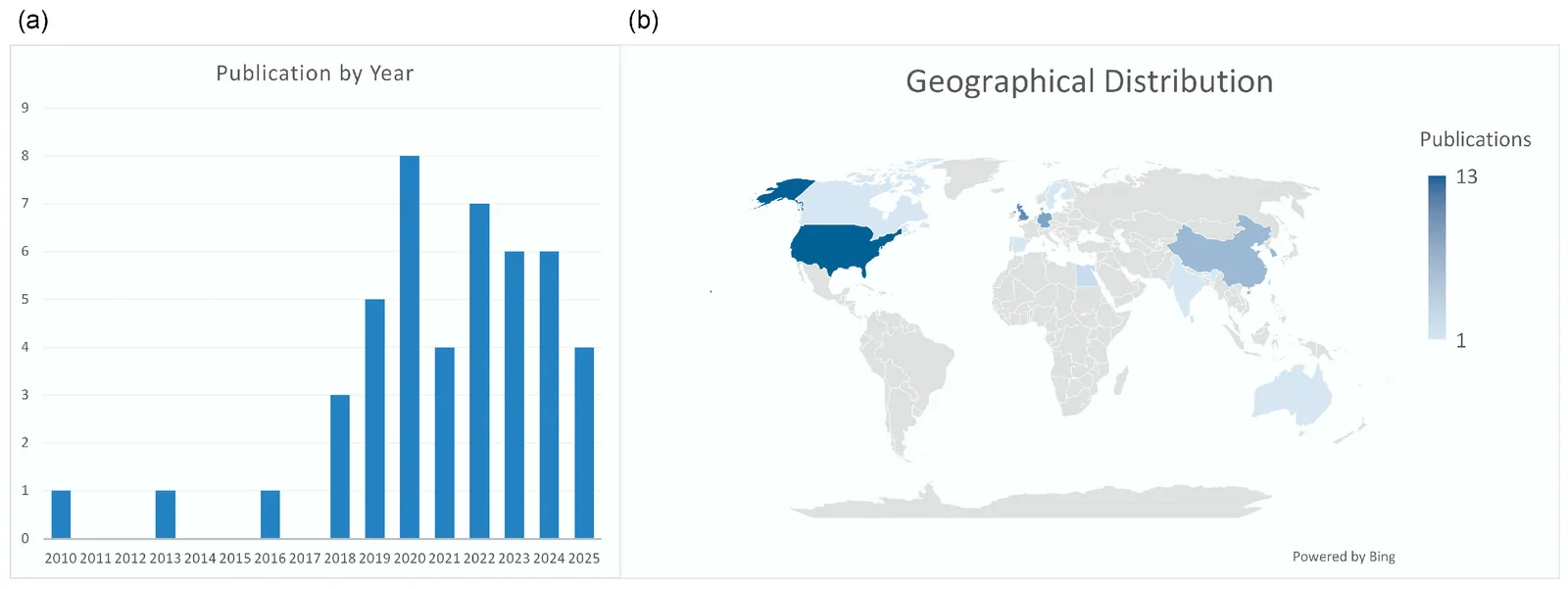
(**a**) Publications per year on AI models for use in hearing devices. (**b**) Geographic distribution of included studies by country of origin (*n* = 46). In panel (**b**), the shade of color indicates the number of publications where at least one author is affiliated with an institution in that country. Studies with authors affiliated with more than one country are counted in each.

Publication activity largely increased over the study period, as shown in Figure 2a. Publication years ranged from 2010 to 2025, with 43 of 46 studies published after 2018. Annual output increased to 5 studies in 2019, peaked at 8 studies in 2020, and remained elevated from 2021 to 2025. The 46 studies were conducted across 18 countries (Figure 2b). The United States contributed to the most studies (*n* = 13, 28.2%), followed by the United Kingdom (*n* = 9, 19.6%) and Germany (*n* = 7, 12.1%) (Supplementary Table S3).

The studies used AI to address several tasks; Table 1 summarizes the primary task accomplished by each study. Speech enhancement was by far the most common task (*n* = 27, 58.7%), followed by denoising (*n* = 3, 6.5%), and then wind-noise suppression, alert-signal detection, and environmental-noise classification (*n* = 2 each, 4.3%). The remaining 10 tasks were addressed by a single study (2.2% each): audio–visual synthesis, auditory attention decoding, individualized auditory-processing enhancement, joint speech-source detection and localization, feedback and occlusion management, adaptive dynamic range optimization, user-driven amplification and gain adjustment, listening-effort prediction from acoustic parameters (LEAP), speech segregation, and adaptive acoustic-environment classification.

DNN was the most common architecture overall (*n* = 16, 34.8%), followed by CNN (*n* = 7, 15.2%), hybrid architectures (*n* = 6, 13.0%), and RNN (*n* = 5, 10.9%). Bayesian approaches (*n* = 3, 6.5%) and traditional ML (*n* = 2, 4.3%) were less common. The following architectures were utilized in a single study (2.2% each): U-Net, DCCRN, GAN, ARN, Wave-U-Net, GCRN, and deep CASA.

**Figure 3:**
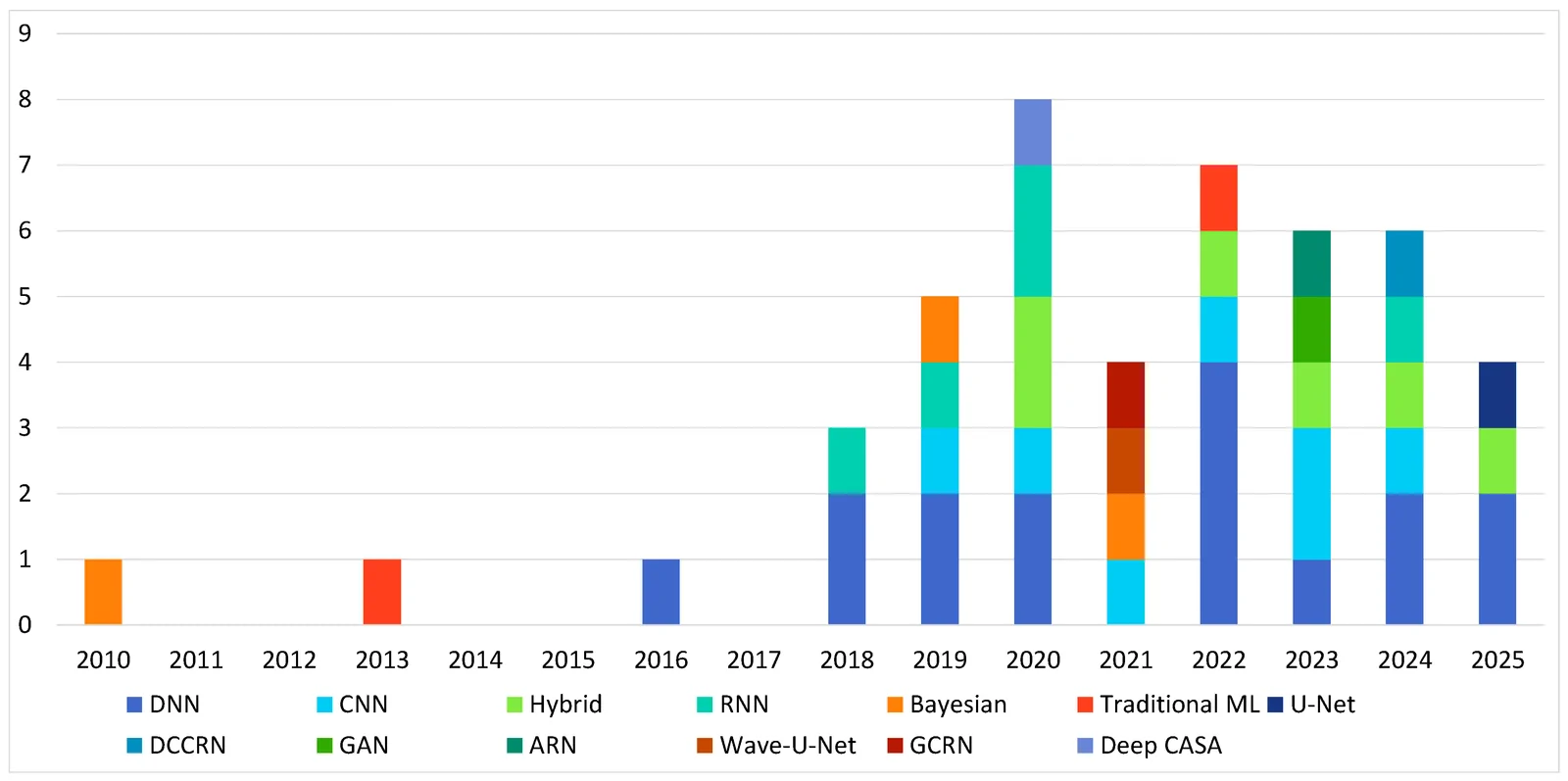
Distribution of AI architecture types by publication year (*n* = 46). Segments represent each architecture’s number of studies within a given year.

Figure 3 demonstrates how model architecture trends have changed over time. DNNs remain the most common architecture overall. Earlier studies include simpler approaches, such as a Bayesian model in 2010 and a traditional ML model in 2014. DNNs were first investigated for use in hearing devices in 2016, and models have implemented DNNs nearly every year after. The number of distinct architectures published per year has generally increased over time; 2021–2025 shows the highest diversity of model architectures, with newer model families (e.g., GAN, ARN, DCCRN, U-Net) appearing only in the most recent years.

**Figure 4:**
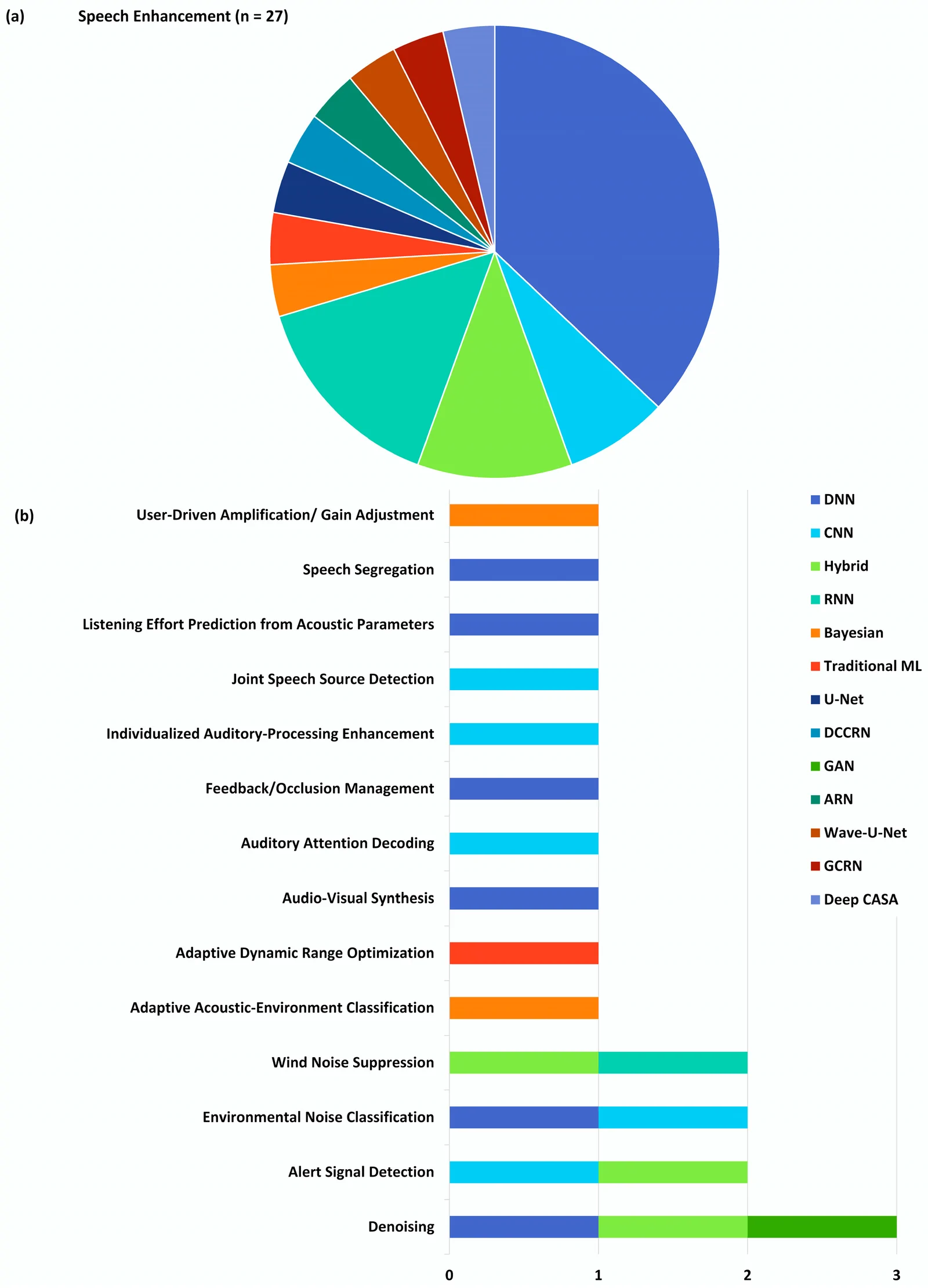
(**a**) Distribution of AI architecture types for speech enhancement (*n* = 27). (**b**) Distribution of AI architecture types by all other primary AI tasks. Segments represent each architecture’s share of studies within a given task.

AI architecture varied by model task; Figure 4 shows the distribution of these architectures across primary tasks. Speech Enhancement is the most common task and includes the widest range of model architectures. Of the models in the speech enhancement category, DNN was the most common (*n* = 10, 37.0%), followed by RNN (*n* = 4, 14.8%), hybrid architectures (*n* = 3, 11.1%), and CNN (*n* = 2, 7.4%). Traditional ML, Bayesian, DCCRN, ARN, U-Net, Wave-U-Net, GCRN, and Deep CASA are all used by a single model for speech enhancement (3.7% each).

The most common model input was sound frequency or spectrogram (*n* = 29, 63.0%). Models also regularly used hybrid inputs (*n* = 7, 15.2%) and time or raw audio (*n* = 6, 13.0%). Two studies used another input type (4.3%), one used none (2.2%), and one was EEG-based (2.2%). For processing site, most models ran on a personal computer (PC) or an offline platform (*n* = 38, 82.6%); six ran on a smartphone (13.0%), and two ran within the hearing aid itself (4.3%).

Table 1 summarizes the learning strategies adopted across studies. AI models were primarily trained using offline (*n* = 43, 93.5%) and supervised (*n* = 42, 91.3%) methods. Five studies used a pre-training phase using a database (10.9%). Additional learning strategies were relatively uncommon: unsupervised learning (*n* = 3, 6.5%), reinforcement learning (*n* = 2, 4.3%), and teacher–student, active learning, contrastive divergence, generative adversarial training, and variational inference (*n* = 1 each, 2.2%).

Table 1 summarizes the acoustic conditions used during testing. Testing most commonly involved speech babble or competing voices (*n* = 27, 58.7%) and steady-state noise (*n* = 26, 56.5%). Real-world, dynamic sound conditions were tested in 16 studies (34.8%), and transient or impulsive noise was tested in 9 studies (19.6%).

Model validation most often used database- or simulation-based testing (*n* = 33, 71.7%), and 26 of the included models (56.5%) were validated by human listeners (Supplementary Table S3). Of those validated by human listeners, 18 were validated by normal-hearing listeners (39.1%), and 17 by hearing-impaired listeners in a lab setting (37.0%). Only 1 model (2.2%) was validated using hearing-impaired human study participants in real-world conditions. An acoustic manikin was used for validation of 3 models (6.5%). One study (2.2%) used normal-hearing listeners with simulated hearing impairment to validate their model.

**Figure 5:**
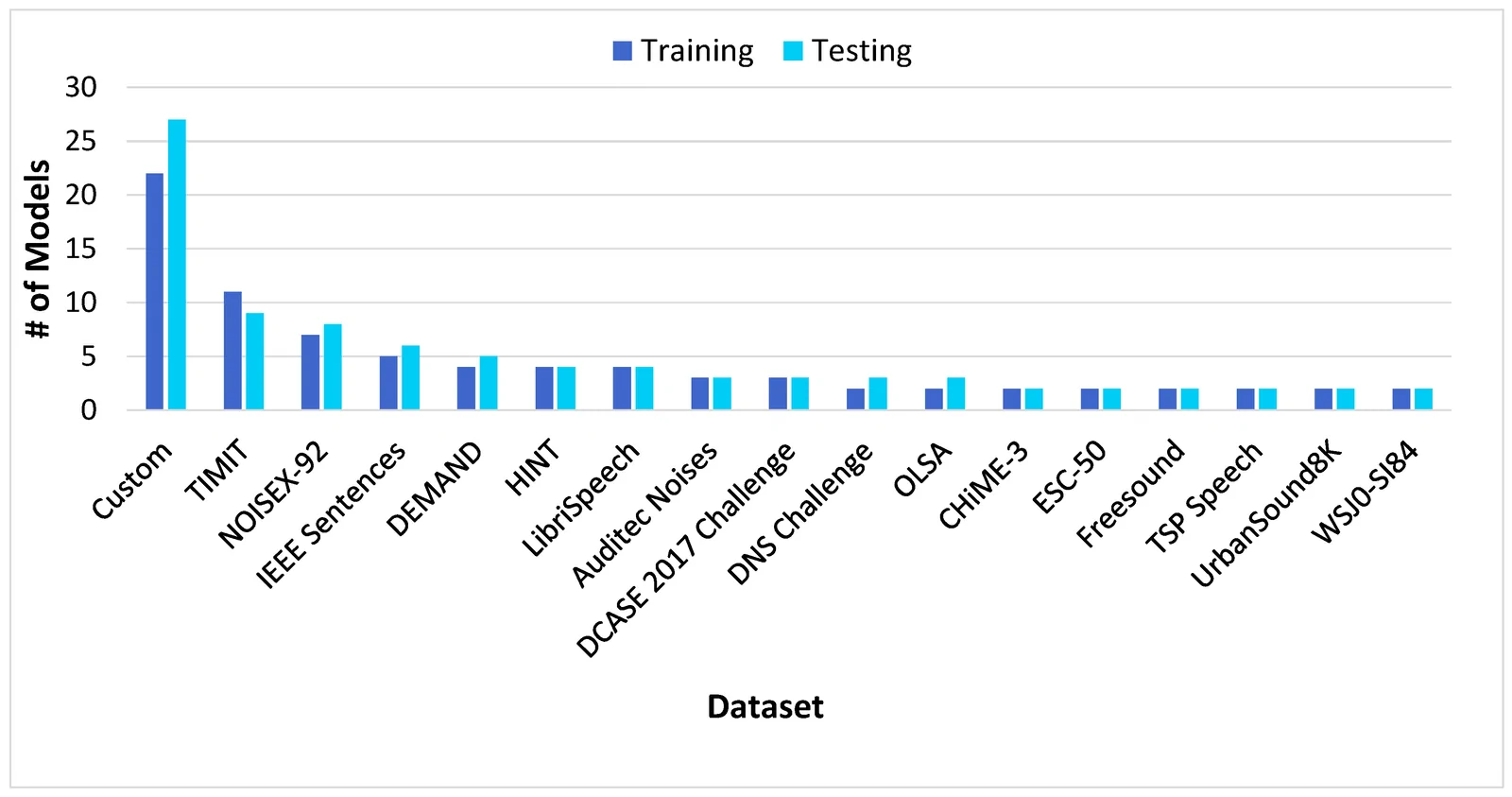
Datasets used for model training and testing. Datasets used exclusively for training or testing and datasets used by only one study were excluded from this graph. The full dataset count is provided in Supplementary Table S3. Definitions of each dataset can be found in Appendix A.

Across the 46 studies, 78 separate datasets, databases, or corpora were used for model training and testing (Supplementary Table S3). Descriptions of each dataset are included in Appendix A. Most studies drew on more than one dataset for each of the phases. The most common training datasets were custom-recorded sounds made by the researchers (47.8%), the TIMIT Acoustic-Phonetic Continuous Speech Corpus (23.9%), and the LibriSpeech Automatic Speech Recognition (ASR) Corpus (5.2%). The most common testing datasets were custom-recorded sounds made by the researchers (58.7%), the TIMIT Acoustic-Phonetic Continuous Speech Corpus (19.6%), and the NOISEX-92 Database (17.4%). The training and testing phases of the different models were highly heterogeneous; 41 training datasets and 46 testing datasets were used to develop a single model (Supplementary Table S3). Figure 5 shows the most frequently used datasets during training and testing.

Each model’s reported or derived algorithmic latency was evaluated against the end-to-end latency required for hearing aid use (5–10 ms). Studies were classified as either compatible with hearing aid latency requirements (“Yes”), compatible only under specific conditions (“Partial/Conditional”), not compatible (“No”), lacking sufficient information to judge compatibility (“Unable to Determine”), or not applicable for hearing aid use (“Not applicable”). Table 2 shows the results, grouped by model architecture.

Overall, only 10 studies (21.7%) described a model with latency low enough to meet hearing aid latency requirements. An additional 7 studies (15.2%) described models that were compatible under certain conditions. Half of all studies (*n* = 23, 50.0%) described models that were not compatible with hearing aid latency requirements, while latency could not be determined in 5 studies (10.9%) and was not applicable in 1 study (2.2%).

Latency compatibility varied substantially by architecture. RNNs had both the highest number and proportion of latency-compatible models (4 of 5 models, 80.0%). Bayesian approaches (2 of 3 models; 66.7%) were mostly compatible, and the single DCCRN model (1 of 1, 100%) met hearing aid latency requirements. DNNs, the most common architecture used by the AI models, met latency requirements in only 2 of 16 studies (12.5%). Half of the DNN models were not compatible with the latency requirements for current use in hearing aids (8 of 16, 50.0%). No CNN model was latency-compatible; CNN models were split between partial/conditional (3 of 7, 42.9%) and not compatible (4 of 7, 57.1%). Hybrid AI architectures were largely not compatible with hearing aid latency requirements (3 of 6, 50.0%); the compatibility of 2 of the hybrid models could not be determined (33.3%). Several architectures represented by a single study were uniformly not compatible with hearing aid latency requirements (all 1 of 1, 100% “No”): U-Net, GAN, ARN, Wave-U-Net, GCRN, and Deep CASA.

The primary task, model architecture, and key findings of each included study are summarized in Table 3. Reported outcomes varied, including objective and subjective metrics. Direct quantitative comparison between studies was not performed due to the heterogeneity between model training methods and reported outcomes.

## 4. Discussion

This scoping review characterized the applications of AI to hearing devices over 15 years, mapping 46 studies by task, architecture, learning approach, input, processing site, validation strategy, and algorithmic latency. Research in hearing devices has grown rapidly; 93.5% of studies were published after 2018. The architectures used in the various models have also increased over the study period. The field is heavily concentrated on speech enhancement, which accounted for 58.7% of studies. DNNs were the most common architecture type (34.8%), and almost every model used supervised, offline learning (91.3% and 93.5%, respectively).

The distinction between algorithmic performance and clinical translation is a central theme. The AI model’s performance on benchmark datasets or objective signal-quality metrics does not necessarily translate to a demonstrated benefit for a hearing-impaired listener on an actual hearing device in real-world listening conditions. The current literature is heavily weighted towards computational algorithmic performance, with 20 of 46 studies lacking validation with human listeners. A total of 71.7% of studies used database and simulation validation, and only a single model was validated by hearing-impaired listeners in real-world conditions. Furthermore, only 2 models were implemented on the hearing device itself. The imbalance between calculated performance and real patient benefit is a major barrier to clinical adoption.

### 4.1. Evolution of AI Architectures

Trends in the underlying AI architecture reflect advances in audio signal processing and AI models. Traditional approaches, like Bayesian and traditional ML, were frequently utilized in the earlier years of the study period [17,18]. Deep learning models were first published for use in hearing devices in 2016 [19]. DNNs subsequently became the most frequently proposed architecture and appear in nearly every publication year after their introduction. The most recent period (2021–2024) experienced both the highest number of publications and the most architecture variety. These recent studies have proposed generative, attention-based, encoder–decoder, and complex recurrent architectures, including GAN, ARN, DCCRN, U-Net, and Wave-U-Net models [47,50,53,54,55,60]. Researchers are actively exploring which model architecture best fits the constraints of wearable auditory devices, rather than anchoring on a single model type.

### 4.2. Concentration on Speech Enhancement and Evidence of Efficacy

Understanding speech in noise remains a major complaint among hearing aid users and is a limitation of conventional noise-reduction systems, so, understandably, research is concentrated on speech enhancement [3]. Traditional noise reduction algorithms have improved listening comfort but have not increased word recognition. Several studies suggest that deep learning approaches may overcome some of the historical limitations of traditional noise reduction algorithms. Bramsløw et al., Healy et al., and Diehl et al. reported significant improvements in speech intelligibility for listeners with hearing loss across a variety of acoustic conditions, including competing talkers, reverberation, and previously untrained noises [3,20,24,31,37,50]. Some of these models achieved these gains under causal or low-latency constraints, showing they are not limited to unrestricted offline use. Diehl et al. further reported that intelligibility for hearing-impaired users could be restored toward normal-hearing levels across steady-state noise, speech babble, and real-world dynamic conditions [3]. Healy et al. described intelligibility improvements of roughly 46–65 percentage points [31,50]. These results represent the strongest evidence from the review that AI-based processing can surpass conventional signal processing in terms of hearing benefit.

### 4.3. Model Heterogeneity and the Limits of Cross-Study Comparison

Direct quantitative comparison across studies was not possible; the heterogeneity between models is itself a principal finding. Studies differed markedly in validation methods, comparator models, reported outcome metrics, and training/testing conditions. The datasets, databases, and corpora used to train the models varied widely; across the 46 studies, 78 distinct datasets were used, and 41 training and 46 testing datasets were each used by only one model. Custom datasets recorded by the studies’ authors were used for training in 22 studies and for testing in 27 studies. Of the studies that used the same datasets for training, the authors often applied reverberation or some other distortion to the sound. The heavy reliance on non-standardized, study-specific data and the lack of standardized metrics make it difficult to compare AI model types across studies. Most models performed well under specific conditions, but their performance generally decreased when exposed to untrained acoustic environments. The aggregate literature shows promise, but most AI models’ results do not generalize to real-world environments.

### 4.4. Underexplored Applications Across the Acoustic Pipeline

Although speech enhancement is the primary application, AI has been applied to a wide range of functions: source localization, feedback and occlusion management, auditory attention decoding, listening effort prediction, audio–visual synthesis, environmental classification, and personalized amplification [21,22,27,28,29,33,38,42,45,49,51,56]. Most of these tasks are represented by only one or two studies, indicating they remain relatively underexplored. Several of these models are notable for addressing needs that speech enhancement or denoising algorithms alone cannot meet: alert signal detection preserves safety-relevant sounds that aggressive denoising models may attenuate [26,28,38]; auditory attention decoding from EEG offers a strategy to steer enhancement towards the talker a listener intends to follow without requiring an explicit acoustic cue [56]; traditional ML models offer real-time individualization of gain and dynamic-range settings [42]. The variety of these applications suggests that AI may eventually support every aspect of the sound-processing chain rather than serving only to help with noise reduction.

### 4.5. The Gap Between Research and Clinical Application

Despite advances in AI algorithms, a discrepancy remains between performance under lab-controlled conditions and real-world performance. This gap has two dimensions: model validation and low-latency requirements.

Database- or simulation-based testing was the most common validation approach, used in 33 of 46 studies (71.7%), and 20 studies included no human validation. Of the 26 studies that used human listeners, most were conducted in lab-based settings: 18 involved normal-hearing listeners and 17 involved hearing-impaired listeners. Only one study (2.2%) evaluated hearing-impaired listeners in real-world conditions, and only one study used ecological momentary assessment to evaluate real-world conditions [52]. Validating a model exclusively with normal-hearing listeners is insufficient to establish its utility for hearing-impaired users because sensorineural hearing loss reduces auditory function in ways beyond reduced audibility. Cochlear damage causes suprathreshold deficits, such as reduced frequency selectivity, impaired coding of temporal fine structure, loudness recruitment with a narrowed dynamic range, and disrupted auditory scene analysis; these deficits persist even with adequate amplification [62,63]. Hearing-impaired listeners also recruit additional cognitive resources like working memory, processing speed, and inhibitory control to decode degraded audio input [64]. Since normal-hearing listeners lack these deficits, performance measured in normal-hearing listeners may overestimate the benefit in hearing-impaired individuals. The lack of real-world evidence in hearing-impaired listeners is therefore the most important barrier to implementing these algorithms in clinical practice.

Hearing aids must process sound with an end-to-end delay of roughly 5 to 10 ms before self-voice coloration, echo, and sound-quality degradation become perceptible [16]. Only 10 of 46 models (21.7%) reported an algorithmic latency low enough to be feasible for current hearing aid use before considering any additional latency from hardware; 7 models (15.2%) were latency-compatible only under specific conditions. Half of all models were not compatible with hearing aid latency requirements. Latency compatibility varied by architecture. RNNs were the most frequently compatible with hearing aid latency requirements (4 of 5; 80.0%), and the single DCCRN was also compatible [54]. The most studied architecture, DNN, met latency requirements in only 2 of 16 studies (12.5%). No CNN model met the latency requirement for device use. Several of the highest-performing enhancement models were also the most computationally demanding. Additionally, 82.6% of models ran on a PC or offline platform, and only 2 (4.3%) were implemented within a hearing device itself. Researchers have rarely tested the memory, power, and real-time constraints of portable hearing devices. More recent studies have tried to close this gap by applying realistic hearing-device constraints during model development. Still, even some of the models that the authors frame as “low latency” do not meet the requirement of 5 to 10 ms [44,54,57,60].

### 4.6. A Framework for Clinical Translation

These findings can be organized as a translational hierarchy through which a model should progress before clinical implementation: (1) database- or simulation-based validation; (2) laboratory testing in normal-hearing listeners; (3) laboratory testing in hearing-impaired listeners; (4) implementation and validation on an embedded hearing device operating within its latency, power, and memory constraints; and (5) validation in hearing-impaired listeners under real-world conditions. A model intended for clinical use should, ultimately, clear all 5 stages. The included studies concentrate almost entirely on the earliest stages of this hierarchy. In total, 33 of 46 studies reported database- or simulation-based validation, 18 normal-hearing laboratory tests, and 17 hearing-impaired laboratory tests. Only 2 studies implemented the AI model on the hearing device itself, and only 1 study reached real-world validation in hearing-impaired listeners. Future work should address the steep attrition between laboratory and real-world application.

### 4.7. Future Directions

Framed against the translational hierarchy above, future research should seek to advance models from simulation and normal-hearing laboratory testing (stages 1–2) to on-device implementation and validation in hearing-impaired listeners under real-world conditions (stages 4–5), the stages at which current evidence is limited. Studies should evaluate AI models in hearing-impaired listeners under realistic acoustic conditions. The most frequently cited limitation among the included studies was reliance on simulated data, objective metrics, and normal-hearing listeners or offline testing [19,23,28,38,44,54,60]. Many models were trained and tested on a narrow range of talkers and noise types [18,24,31,40,41,49]; they should be tested in dynamic, reverberant, moving-source scenes with listener head movement in the future.

Additionally, the current models require too much computational power or have too high latency to run on hearing aid hardware. Future studies should focus on model compression and reducing computational/memory footprints. To increase the number of models that can run on hearing aid hardware, researchers should consistently measure each model’s algorithmic and end-to-end latency, power, and energy requirements. Included studies commonly reported Bluetooth connectivity as a barrier to clinical implementation. One study reported Bluetooth latency of around 440 ms, making smartphone-linked processing unsuitable for real-time conversations [32]. Future directions include concurrently designing AI models with dedicated hardware that can handle the computational requirements of larger models and converting non-causal architectures to causal forms.

### 4.8. Limitations

This review has several limitations. As a scoping review, it was designed to map the breadth and characteristics of the literature rather than formally assess study quality or pool effect sizes. Heterogeneity across model training and testing phases precluded quantitative synthesis or comparisons. The inclusion criteria limited the review to English-language, original, peer-reviewed research published between 2010 and August 2025. This approach may have introduced publication bias by excluding relevant work in other languages, conference abstracts, industry white papers, and other non-peer-reviewed literature. Commercial proprietary algorithms that were not published were also excluded. Additionally, latency feasibility was assessed against a single 5 to 10 ms target, and some studies did not report latency, introducing a degree of judgment into the latency classification.

## 5. Conclusions

To map AI applications in hearing devices, this scoping review examined 46 studies that met the inclusion criteria. The evidence base is expanding rapidly, especially within the last 5–8 years. The architecture types have evolved from traditional ML and Bayesian models to a larger variety of more complex deep learning architectures. The field remains heavily concentrated on speech enhancement, but AI models have been proposed for a variety of sound-processing functions.

Most models were developed and evaluated under laboratory conditions; model training was largely offline and supervised. Model testing and validation mostly relied on database- or simulation-based evaluations; studies rarely tested algorithms with hearing-impaired listeners under real-world conditions or on the actual hearing device. Only 10 of the 46 studies met the low-latency requirements for real-world implementation on hearing aids, and latency compatibility varied across AI architectures. Because of heterogeneity between studies, the models could not be directly compared across studies, and this review is unable to make any claims about the comparative efficacy of individual models.

AI has been applied across nearly every aspect of sound processing in hearing devices and has shown promising results. However, most of the current literature consists of model development or proof-of-concept studies. Only a minority of studies evaluate the AI model on a hearing device or with hearing aid constraints. Widespread implementation will require prospective evaluation in target populations and real-world environments. Addressing these priorities would help close the gap between early algorithm development and clinical application.

## Figures and Tables

**Table 1:** Descriptive Characteristics.

Characteristic	Category	*n* (%)
N	Total Study Number	46 (100)
Study Type	Model Development and Validation	41 (89.1)
	Laboratory Perceptual Evaluation	4 (8.7)
	Real-World Effectiveness	1 (2.2)
Narrative Fit	Engineering	25 (54.3)
	Translational	21 (45.7)
Primary Task	Speech Enhancement	27 (58.7)
	Denoising	3 (6.5)
	Wind Noise Suppression	2 (4.3)
	Alert Signal Detection	2 (4.3)
	Environmental Noise Classification	2 (4.3)
	Audio–Visual Synthesis	1 (2.2)
	Auditory Attention Decoding	1 (2.2)
	Individualized Auditory-Processing Enhancement	1 (2.2)
	Joint Speech Source Detection/Localization	1 (2.2)
	Feedback/Occlusion Management	1 (2.2)
	Adaptive Dynamic Range Optimization (ADRO)	1 (2.2)
	User-Driven Amplification/Gain Adjustment	1 (2.2)
	Listening Effort Prediction from Acoustic Parameters (LEAP)	1 (2.2)
	Speech Segregation	1 (2.2)
	Adaptive Acoustic-Environment Classification	1 (2.2)
AI Model Type	Deep Neural Network (DNN)	16 (34.8)
	Convolutional Neural Network (CNN)	7 (15.2)
	Hybrid	6 (13.0)
	Recurrent Neural Network (RNN)	5 (10.9)
	Bayesian	3 (6.5)
	Traditional Machine Learning (ML)	2 (4.3)
	U-Net	1 (2.2)
	Deep Complex Convolutional Recurrent Network (DCCRN)	1 (2.2)
	Generative Adversarial Network (GAN)	1 (2.2)
	Attentive Recurrent Network (ARN)	1 (2.2)
	Wave-U-Net	1 (2.2)
	Gated Convolutional Recurrent Network (GCRN)	1 (2.2)
	Deep Computational Auditory Scene Analysis (Deep CASA)	1 (2.2)
Learning Method	Offline	43 (93.5)
	Supervised	42 (91.3)
	Pre-Training Phase with Database	5 (10.9)
	Unsupervised	3 (6.5)
	Reinforcement	2 (4.3)
	Teacher–Student	1 (2.2)
	Active Learning	1 (2.2)
	Contrastive Divergence	1 (2.2)
	Generative Adversarial Training	1 (2.2)
	Variational Interference	1 (2.2)
Model Input	Frequency (Spectrogram)	29 (63.0)
	Hybrid	7 (15.2)
	Time (Raw Audio)	6 (13.0)
	Other	2 (4.3)
	None	1 (2.2)
	EEG-Based	1 (2.2)
Processing Site	PC/Offline	38 (82.6)
	Smartphone	6 (13.0)
	On-chip (within Hearing Device)	2 (4.3)
Acoustic Testing Environment	Speech Babble/Competing Voices	27 (58.7)
	Steady-State Noise	26 (56.5)
	Real-World/Dynamic	16 (34.8)
	Transient/Impulsive	9 (19.6)
Validation Method	Database/Simulation	33 (71.7)
	Human: Normal-Hearing	18 (39.1)
	Human: Hearing-Impaired (Lab)	17 (37.0)
	Acoustic Manikin	3 (6.5)
	Human: Hearing-Impaired (Real World)	1 (2.2)
	Human: Simulated Hearing-Impaired	1 (2.2)

**Table 2:** Compatibility of AI model architectures with hearing aid latency requirements relative to the target latency range of 5 to 10 ms.

AI Architecture	Yes (%)	Partial/ Conditional (%)	No (%)	Unable to Determine (%)	Not Applicable (%)	Total
DNN	2 (12.5)	2 (12.5)	8 (50.0)	3 (18.8)	1 (6.3)	16
CNN	0 (0.0)	3 (42.9)	4 (57.1)	0 (0.0)	0 (0.0)	7
Hybrid	0 (0.0)	1 (16.7)	3 (50.0)	2 (33.3)	0 (0.0)	6
RNN	4 (80.0)	0 (0.0)	1 (20.0)	0 (0.0)	0 (0.0)	5
Bayesian	2 (66.7)	0 (0.0)	1 (33.3)	0 (0.0)	0 (0.0)	3
Traditional ML	1 (50.0)	1 (50.0)	0 (0.0)	0 (0.0)	0 (0.0)	2
ARN	0 (0.0)	0 (0.0)	1 (100.0)	0 (0.0)	0 (0.0)	1
DCCRN	1 (100.0)	0 (0.0)	0 (0.0)	0 (0.0)	0 (0.0)	1
Deep CASA	0 (0.0)	0 (0.0)	1 (100.0)	0 (0.0)	0 (0.0)	1
GCRN	0 (0.0)	0 (0.0)	1 (100.0)	0 (0.0)	0 (0.0)	1
GAN	0 (0.0)	0 (0.0)	1 (100.0)	0 (0.0)	0 (0.0)	1
U-Net	0 (0.0)	0 (0.0)	1 (100.0)	0 (0.0)	0 (0.0)	1
Wave-U-Net	0 (0.0)	0 (0.0)	1 (100.0)	0 (0.0)	0 (0.0)	1
Total	10 (21.7)	7 (15.2)	23 (50.0)	5 (10.9)	1 (2.2)	46

Abbreviations: DNN, Deep Neural Network; CNN, Convolutional Neural Network; RNN, Recurrent Neural Network; ML, Machine Learning; ARN, Attentive Recurrent Network; DCCRN, Deep Complex Convolutional Recurrent Network; Deep CASA, Deep Computational Auditory Scene Analysis; GCRN, Gated Convolutional Recurrent Network; GAN, Generative Adversarial Network.

**Table 3:** Main outcomes and findings of included studies.

Author, Year (Journal)	Model Architecture	Primary Task	Key Findings
Lemarche, 2010 [17]	Bayesian	Adaptive Acoustic- Environment Classification	A fully unsupervised adaptive Bayesian classifier autonomously split, merged, and tuned acoustic-environment classes during use. The Bayesian classifier outperformed a minimum-distance classifier and approached supervised accuracy.
Ayllón, 2013 [18]	Traditional ML	Speech Enhancement	A binaural ML algorithm using ideal binary mask time-frequency masking enabled bilateral hearing aids to exchange information using <10% of processor resources at bit rates < 64 kbps. The model separated speech from noise across multiple environments and outperformed a monaural approach.
Chen, 2016 [19]	DNN	Speech Segregation	A DNN trained on 10,000 diverse noises generalized to novel noises and untrained SNRs, resulting in significant speech-intelligibility gains for hearing-impaired listeners.
Bramsløw, 2018 [20]	DNN	Speech Enhancement	Low-latency (8 ms) DNN improved competing talker segregation in 15 hearing-impaired listeners (~13-point benefit for divided attention; ~37-point benefit for single target).
Huber, 2018 [21]	DNN	LEAP	A single-ended DNN predicted perceived listening effort from noisy or processed speech (correlations > 0.9) without a clean reference or transcript, outperforming conventional single-ended quality metrics.
Keshavarzi, 2018 [22]	RNN	Wind Noise Suppression	A deep LSTM RNN estimated an ideal ratio mask to reduce wind noise in behind-the-ear recordings; processed speech was modestly but significantly preferred over unprocessed speech for intelligibility and quality in both normal-hearing and hearing-impaired listeners.
Bhat, 2019 [23]	CNN	Speech Enhancement	A compact multi-objective CNN (LPS + Log Mel-filterbank targets) that performed real-time, single-microphone smartphone speech enhancement (~14 ms latency) with no external hardware. The CNN model generally outperformed conventional and neural baselines on objective and subjective metrics for hearing aid users.
Healy, 2019 [24]	RNN	Speech Enhancement	A single-microphone RNN-BLSTM estimated the ideal ratio mask. It de-reverberated and separated a target from a single competing talker plus reverberation, producing large intelligibility and quality gains. Direct-sound (reverberation-removing) processing was superior for hearing-impaired listeners.
Lai, 2019 [25]	DNN	Speech Enhancement	Adding multi-objective learning (joint LPS and MFCC targets) to a DNN model improved speech quality and maintained intelligibility over single-objective deep learning and conventional speech enhancement. The largest improvements were in fast-changing noise and at low SNRs.
Nossier, 2019 [26]	DNN	Speech Enhancement	DNN-based speech enhancement model selectively retained safety-relevant alerting sounds (such as fire alarms and car horns) while suppressing unwanted noise.
Søgaard Jensen, 2019 [27]	Bayesian	User-Driven Amplification/Gain Adjustment	The SoundSense Learn (app-based machine learning) personalization method significantly improved basic audio quality and increased listening comfort in traffic noise. The model did not improve speech clarity. Benefits and preferred gain settings varied by individual and were not predicted by setting changes or audiograms.
Bhat, 2020 [28]	Hybrid	Alert Signal Detection	This study described a real-time smartphone hearing aid pipeline that added a CNN alert-signal detector and a CRNN (stacked-GRU) ahead of the speech enhancement/compression stage. The system detected warning sounds, flagged them on-screen, and separated them so they were preserved rather than attenuated while suppressing background noise. The model ran on device with about 5 ms of processing latency.
Fan, 2020 [29]	DNN	Environmental Noise Classification	A smartphone-deployed DNN using stacked spectral audio features and majority voting across sequential blocks classified five everyday sound environments with up to 98.8% accuracy, offering a potential front-end for automatic hearing aid program and gain adjustment while offloading computation from the hearing device.
Gogate, 2020 [30]	DNN	Speech Enhancement	A causal, audio–visual DNN using lip cues alongside noisy audio meaningfully improved speech quality and intelligibility over audio-only and conventional methods, especially in severe real-world noise. The model generalized across speakers, vocabularies, and a new language without retraining.
Healy, 2020 [31]	Deep CASA	Speech Enhancement	A single-microphone, talker-independent deep learning algorithm (deep CASA) can produce large, consistent speech-intelligibility gains (~65 percentage points) for hearing-impaired listeners in competing talkers plus substantial room reverberation. The model generalized to conditions not seen during training.
Li, 2020 [32]	RNN	Speech Enhancement	A GRU-based RNN binaural speech enhancement algorithm offloaded to a smartphone app improved objective speech-quality and intelligibility metrics better than conventional and prior deep-learning methods.
Park, 2020 [33]	CNN	Environmental Noise Classification	A spectrogram-based CNN can classify everyday environmental noise for hearing aids with very high internal accuracy (up to about 99%). Pairing image pre-processing filters with longer analysis windows preserved accuracy while reducing the number of classifications needed over time.
Park, 2020 [34]	Hybrid	Speech Enhancement	The model used CNN acoustic environment classification followed by a noise-specific pre-trained DNN denoiser. The results showed consistent objective improvements in speech quality and intelligibility compared with a single unclassified DNN and a conventional hearing aid algorithm.
Shankar, 2020 [35]	RNN	Speech Enhancement	A compact two-microphone RNN using real and imaginary STFT features improved speech quality and intelligibility to levels comparable to a more complex LSTM network, at a much lower computational cost.
Fischer, 2021 [36]	Wave-U-Net	Speech Enhancement	This study is a proof of concept for the MVDR Beamforming algorithm, where increasing the number of microphones spatially across the head and closer to the target enhances speech in noise. A Wave U-Net processed these virtual microphone signals and fed them to an MVDR beamformer, improving objective speech quality and user preference in noisy environments.
Healy, 2021 [37]	GCRN	Speech Enhancement	A causal, single-microphone GCCRN algorithm improved speech intelligibility for hearing-impaired listeners in previously unseen, complex real-world noise environments while maintaining latency compatible with hearing aid use. The largest improvements occurred under the most challenging acoustic conditions and in listeners with greater hearing loss.
Kang, 2021 [38]	CNN	Alert Signal Detection	A parallel CNN accurately classified warning sounds within noisy speech (roughly 98% to 99% accuracy) and was paired with a CEDN to enhance both speech and warning signals.
van Erp, 2021 [39]	Bayesian	Speech Enhancement	A modular Bayesian generative-model framework personalized noise reduction in real time and improved objective speech-quality and intelligibility metrics.
Green, 2022 [40]	DNN	Speech Enhancement	Combining beamformers with a DNN-based speech enhancement model did not improve performance compared to beamforming alone.
Kayser, 2022 [41]	DNN	Speech Enhancement	Probabilistic DOA localized speech reliably in diffuse noise but failed against a dominant localized non-speech interferer. Combining probabilistic DOA with a reference-free DNN phoneme-based speech-quality measure restored correct target-source selection in most conditions and enabled blind beamformer steering.
Ni, 2022 [42]	Traditional ML	ADRO	An online MLIRL method personalized ADRO hearing aid comfort targets from listener preferences, producing a fit that was strongly preferred over standard ADRO (roughly 10:1) while maintaining or slightly improving speech recognition.
Nossier, 2022 [43]	Hybrid	Speech Enhancement	This proof-of-concept engineering study showed that a DNN speech enhancement plus CNN noise-classification system enabled an environmentally adaptable hearing aid; overall classification accuracy was modest at 70%.
Schröter, 2022 [44]	DNN	Speech Enhancement	A low-latency (8 ms), low-complexity two-stage DNN combined deep filtering and a novel frequency-response loss matched state-of-the-art deep learning speech enhancement on objective metrics. The proposed DNN model also outperformed a conventional hearing aid noise reduction algorithm in intelligibility and perceptual preference.
Shan, 2022 [45]	DNN	Audio–Visual Synthesis	A DNN-based face-synthesis model generated a visual (synthesized face) from an acoustic signal to accompany speech cues, meaningfully improving speech-in-noise comprehension.
Shifas, 2022 [46]	CNN	Speech Enhancement	A single end-to-end neural network operating on raw noisy audio performed noise reduction and near-end intelligibility enhancement. The model improved objective and listener-based measures compared with conventional signal-processing methods.
Cheng, 2023 [47]	GAN	Denoising	Compact, fully causal FTCRN metric-GAN was developed for joint denoising and audiogram-based hearing-loss compensation; the model demonstrated the highest HASQI vs. comparators (behind DB-AIAT on WB-PESQ/STOI).
Diehl, 2023 [3]	Hybrid	Denoising	This model improved speech-in-noise intelligibility for hearing-impaired users to levels comparable to those of normal-hearing listeners across steady-state noise, speech babble, and real-world dynamic noise.
Drakopoulos, 2023 [48]	CNN	Individualized Auditory-Processing Enhancement	A simulation-based proof of concept in which a differentiable, biophysically grounded neural-network framework trained individualized end-to-end hearing aid models to restore simulated auditory-nerve activity for outer hair cell loss. The model matched or exceeded conventional amplification, but only partially compensated for cochlear synaptopathy.
Goli, 2023 [49]	CNN	Joint Speech Source Detection and Localization	This paper proposed a CNN that enabled accurate speech detection and localization in noisy, reverberant conditions using hearing aid microphone geometry. The model outperformed STFT-based and conventional GCC-PHAT approaches and generalized to unseen room sizes/head orientations.
Healy, 2023 [50]	ARN	Speech Enhancement	A fully causal deep-learning enhancement model generalized across untrained talkers, corpora, and noise types. The model produced large laboratory speech-in-noise intelligibility gains of about 46% to 58% for hearing-impaired listeners under real-time constraints.
Zheng, 2023 [51]	DNN	Feedback/Occlusion Management	Across the three DNN-based model variations tested on speech and music, pairing DeepMFC with PEM-AFC gave >13 dB additional stable gain in simulated hearing-impaired listeners. The combination DeepMFC + PEM-AFC was subjectively preferred at −5/−10 dB gain margins.
Christensen, 2024 [52]	DNN	Denoising	In real-world use, DNN-based noise reduction produced consistent sound satisfaction regardless of background-noise level compared to an SNR-dependent traditional statistical noise reducer. Both models preserved ambient awareness.
Esra, 2024 [53]	DNN	Speech Enhancement	The DNN was an attention-masking separation optimized by a novel Fitness-Ordered Black Widow Optimization metaheuristic. The model performed joint speaker separation and noise separation; it was designed to separate a single noisy mixture into two speaker streams. Across three benchmark datasets, the author’s model outperformed all comparators on PSNR, PESQ, RMSE, and accuracy.
Kim, 2024 [54]	DCCRN	Speech Enhancement	A DCCRN integrated directly into a low-delay polyphase DFT filterbank delivered meaningful objective speech enhancement gains while operating within the latency and memory constraints of monaural hearing aids. The DCCRN model showed smaller improvements compared to larger AI models.
Lin, 2024 [55]	Hybrid	Wind Noise Suppression	In a controlled bench setting, pairing a redesigned slanted microphone enclosure with wind-classification-driven adaptive beamforming, WDRC, and U-Net deep learning reduced wind noise and substantially improved objective speech intelligibility over conventional high-pass filtering in behind-the-ear hearing aids.
Tanveer, 2024 [56]	CNN	Auditory Attention Decoding	A deep CNN successfully decoded auditory attention direction, speech-versus-noise presence, and hearing aid noise reduction status from short 1 s EEG windows in hearing-impaired listeners. The model completed this task without an acoustic speech signal.
Westhausen, 2024 [57]	RNN	Speech Enhancement	A low-latency, low-complexity DNN improved speech intelligibility for hearing-impaired listeners compared with unprocessed audio. Binaural deep processing showed the largest advantage over conventional algorithms when interfering speech was spatially localized; in diffuse noise, it performed similarly to established directional processing.
Anwar, 2025 [58]	Hybrid	Speech Enhancement	An audio–visual framework integrating a Sugeno neuro-fuzzy model with openMHA adaptively scaled speech enhancement strength using RF-sensed cognitive load, audio SNR, and visual cues. The model ran in near real time on a Raspberry Pi and outperformed audio-only processing.
Overby, 2025 [59]	DNN	Speech Enhancement	In experienced hearing aid users with moderate-to-severe hearing loss, adding a DNN-based noise reduction stage before compression consistently improved subjective listening preference, and pairing it with scene-aware or slow-acting compression approached the preference of an idealized system.
Park, 2025 [60]	U-Net	Speech Enhancement	This model was co-optimized with its hardware to run in real time at 740 µW digital-core power, and it improved objective and subjective speech quality over prior speech enhancement processor designs.
Zhang, 2025 [61]	DNN	Speech Enhancement	A two-stage, low-complexity deep-learning network using source-independent compression performed noise reduction and wide-dynamic-range compression simultaneously. The model outperformed conventional and SNR-aware compression, especially in non-stationary noise.

Abbreviations: µW, microwatts; ADRO, Adaptive Dynamic Range Optimization; ARN, Attentive Recurrent Network; BLSTM, Bidirectional Long Short-Term Memory; CEDN, Convolution Encoder–Decoder Network; CNN, Convolutional Neural Network; CRNN, Convolutional-Recurrent Neural Network; DB-AIAT, Dual-Branch Attention-in-Attention Transformer; DCCRN, Deep Complex Convolutional Recurrent Network; Deep CASA, Deep Computational Auditory Scene Analysis; DeepMFC, Deep Marginal Feedback Cancellation; DFT, Discrete Fourier Transform; DNN, Deep Neural Network; DOA, Direction of Arrival; EEG, Electroencephalogram; FTCRN, Frequency–Time Convolutional Recurrent Network; GAN, Generative Adversarial Network; GCC-PHAT, Generalized Cross-Correlation with Phase Transform; GCRN, Gated Convolutional Recurrent Network; GRU, Gated Recurrent Units; HASQI, Hearing Aid Speech Quality Index; kbps, kilobits per second; LEAP, Listening Effort Prediction from Acoustic Parameters; LPS, Log Power Spectra; LS-GDA, Least Squares Generalized Discrimination Analysis; LSTM, Long Short-Term Memory; MFCC, Mel-Frequency Cepstral Coefficient; ML, Machine Learning; MLIRL, Maximum Likelihood Inverse Reinforcement Learning; ms, milliseconds; MVDR, Minimum Variance Distortionless Response; openMHA, Open Master Hearing Aid; PEM-AFC, Prediction Error Method with Adaptive Feedback Cancellation; PESQ, Perceptual Evaluation of Speech Quality; PSNR, Peak Signal-to-Noise Ratio; RF, Radio Frequency; RMSE, Root Mean Square Error; RNN, Recurrent Neural Network; SNR, Speech–Noise Ratio; STFT, Short-Term Fourier Transform; STOI, Short-Time Objective Intelligibility; WB-PESQ, Wideband Perceptual Evaluation of Speech Quality; WDRC, Wide Dynamic Range Compression.

## Data Availability

There was no new data created or analyzed in this study. Data sharing is not applicable to this article.

## Supplementary Materials

The following supporting information can be downloaded at https://www.mdpi.com/article/10.3390/jcm15208124/s1: Table S1: Preferred Reporting Items for Systematic Reviews and Meta-Analyses extension for Scoping Reviews (PRISMA-ScR) Checklist; Table S2: Electronic Database Search Strategy; Table S3: Complete Dataset of Included Studies.

## Abbreviations

The following abbreviations are used in this manuscript:

µW	Microwatts
ADRO	Adaptive Dynamic Range Optimization
AI	Artificial Intelligence
ARN	Attentive Recurrent Network
ASR	Automatic Speech Recognition
BLSTM	Bidirectional Long Short-Term Memory
CEDN	Convolution Encoder–Decoder Network
CNN	Convolutional Neural Network
CRNN	Convolutional-Recurrent Neural Network
dB	Decibels
DB-AIAT	Dual-Branch Attention-in-Attention Transformer
DCCRN	Deep Complex Convolutional Recurrent Network
Deep CASA	Deep Computational Auditory Scene Analysis
DeepMFC	Deep Marginal Feedback Cancellation
DFT	Discrete Fourier Transform
DNN	Deep Neural Network
DOA	Direction of Arrival
EEG	Electroencephalogram
FTCRN	Frequency–Time Convolutional Recurrent Network
GAN	Generative Adversarial Network
GCC-PHAT	Generalized Cross-Correlation with Phase Transform
GCRN	Gated Convolutional Recurrent Network
GRU	Gated Recurrent Units
HASQI	Hearing Aid Speech Quality Index
kbps	kilobits per second
LEAP	Listening Effort Prediction from Acoustic Parameters
LPS	Log Power Spectra
LS-GDA	Least Squares Generalized Discrimination Analysis
LSTM	Long Short-Term Memory
MFCC	Mel-Frequency Cepstral Coefficient
ML	Machine Learning
MLIRL	Maximum Likelihood Inverse Reinforcement Learning
ms	milliseconds
MVDR	Minimum Variance Distortionless Response
openMHA	Open Master Hearing Aid
PC	Personal Computer
PEM-AFC	Prediction Error Method with Adaptive Feedback Cancellation
PESQ	Perceptual Evaluation of Speech Quality
PRISMA-ScR	Preferred Reporting Items for Systematic Reviews and Meta-Analyses extension for Scoping Reviews
PSNR	Peak Signal-to-Noise Ratio
RF	Radio Frequency
RMSE	Root Mean Square Error
RNN	Recurrent Neural Network
SNR	Speech–Noise Ratio
STFT	Short-Term Fourier Transform
STOI	Short-Time Objective Intelligibility
WB-PESQ	Wideband Perceptual Evaluation of Speech Quality
WDRC	Wide Dynamic Range Compression

## Appendix A. Training and Testing Datasets

100 Nonspeech Environmental Sounds Dataset: Collection of 100 nonspeech environmental sound recordings compiled by Guoning Hu and colleagues for speech enhancement and auditory research.

104 Noise Types (Xu et al.): Set of 104 noise types used in Xu et al.’s DNN speech enhancement work, consisting of 100 environmental noises plus AWGN, babble, restaurant, and street noise [17].

A Wrinkle in Time Audiobook: Audiobook recording of *A Wrinkle in Time* used as competing speech or masker material in speech-in-noise experiments [35].

Additive White Gaussian Noise (AWGN): Synthetic noise signal with a Gaussian amplitude distribution [40].

ASPIRE Dataset: Audiovisual speech corpus recorded in noisy environments, including cafés and restaurants [30].

Audio Source Separation (ASS) Dataset: A publicly available audio source-separation dataset [53].

AudioSet: Large-scale collection of audio clips extracted from YouTube videos, covering speech, music, environmental sounds, human sounds, animals, and other sound events [3].

Auditec Noises: Commercially distributed audiology recordings containing noise materials such as multitalker babble and cafeteria noise [19,37,47].

AVSpeech Dataset: Large-scale audiovisual speech dataset containing 3 to 10 s video clips where audible speech comes from a single visible speaker. It contains approximately 4700 h from approximately 290,000 YouTube videos [3].

BBC Sound Effects Library: Commercial collection of professionally recorded sound effects and environmental or ambient recordings [3,22].

Bern Cocktail Party (BCP) Dataset: Multichannel acoustic dataset containing recordings of target speech, competing speakers, and background noise using multiple microphones mounted on a head-and-torso simulator [36].

Binaural Dataset (Richard et al.): Dataset containing binaural speech data associated with multiple subjects [53].

BKwHA HRTF Dataset: Head-related transfer function database measured using a Brüel & Kjær head-and-torso simulator with hearing aid microphones [49].

CHiME-2 Challenge Dataset: Noisy speech corpus from the second CHiME Speech Separation and Recognition Challenge; it contains WSJ0 speech mixed with reverberant domestic or living-room background environments [33].

CHiME-3 Challenge Dataset: Speech corpus containing real and simulated noisy WSJ0 speech recordings collected or simulated in four real-world environments: bus, café, pedestrian area, and street [30,61].

CIPIC HRTF Database: Public-domain database of head-related impulse responses for 45 subjects, consisting of 43 human subjects and 2 dummy-head subjects, measured at 1250 spatial directions per subject [18].

Clarity Enhancement Challenge (CEC) Dataset: Data associated with the Clarity Enhancement Challenge, a hearing aid speech-in-noise challenge involving simulated acoustic scenes and hearing aid processing [58].

Common Phone Dataset: Multilingual speech corpus derived from Mozilla Common Voice, containing approximately 116 h of speech with automatically generated phonetic segmentation and phonetic annotations [45].

Common Voice Dataset: Large multilingual crowdsourced speech corpus where volunteers contribute and validate recorded speech samples [3,45].

CSTR VCTK Corpus: English multispeaker speech corpus containing recordings from approximately 110 speakers with a range of English accents. Each speaker reads approximately 400 sentences [42].

CUNY Sentence Lists: Speech-testing materials developed at the City University of New York consisting of 60 lists of 12 sentences [54].

Danish Hearing in Noise Test (HINT) Database: Danish version of the Hearing in Noise Test sentence corpus consisting of standardized sentence materials [20].

DCASE 2017 Challenge Dataset: Dataset released for the DCASE 2017 acoustic scene classification challenge, specifically the TUT Acoustic Scenes 2017 dataset. It contains recordings representing 15 real-world acoustic scene classes [23,28,46].

Deep Noise Suppression (DNS) Challenge Dataset: Large speech enhancement corpus released for the Microsoft Deep Noise Suppression Challenge, containing clean speech and diverse noise recordings [45,47,51,57,58,61].

DEMAND (Diverse Environments Multichannel Acoustic Noise Database): Multichannel database of real-world environmental noise recordings collected in diverse indoor and outdoor environments, including domestic, office, transportation, public, and outdoor settings [3,27,58,60,61].

Donate-a-Cry Corpus: Crowdsourced corpus of infant-cry audio recordings collected through the Donate-a-Cry campaign [43].

ESC-50 Dataset: Environmental Sound Classification dataset containing 2000 labeled five-second environmental audio recordings organized into 50 classes [43,59].

ETSI Binaural Background Noise Database: Standardized binaural background-noise material associated with ETSI hearing aid testing and simulation procedures, designed to reproduce realistic background acoustic environments [55].

First Clarity Enhancement Challenge (CEC1) Dataset: Dataset released for the first Clarity Enhancement Challenge [57].

Flemish Matrix Sentence Test: Dutch/Flemish matrix speech corpus consisting of 13 lists of 20 five-word sentences. Each sentence follows a fixed structure using a name, verb, numeral, color, and object selected from a closed vocabulary [48].

Freesound Dataset/Repository: Open online repository of user-contributed sound recordings covering a broad range of environmental, everyday, musical, and other audio material [49,58].

FSDnoisy18k Dataset: Audio dataset that contains 18,532 audio clips totaling approximately 42.5 h across 20 sound classes, with clean and noisy-label portions derived from Freesound [39].

Greek Harvard (GrHarvard) Corpus: Greek-language adaptation of Harvard/IEEE-style phonetically balanced sentence materials [27].

GRID Corpus: Large audiovisual speech corpus containing 1000 structured six-word command sentences from each of 34 speakers, for a total of 34,000 sentences, with synchronized audio, facial video, and word-level transcriptions [30].

GRID-CHiME3 Dataset: Hybrid speech/noise dataset combining clean audiovisual GRID speech with CHiME-3 environmental noise [58].

Hearing in Noise Test (HINT) Database: Standardized speech-testing corpus consisting of sentence materials designed to measure speech reception thresholds in quiet and noise [23,28,46,50].

ICRA Noise Database: Set of artificially generated noise signals developed for hearing aid assessment, with speech-like spectral and temporal characteristics [21,22].

IEEE Sentences Corpus (Harvard Sentences): Standardized corpus of 720 phonetically balanced English sentences, organized into lists of 10 sentences [17,19,24,31,37,40].

ILSAP Database: Greek speech database produced by the Institute for Language and Speech Processing, containing recorded Greek speech material [27].

International Speech Test Signal (ISTS): Standardized speech-like test signal constructed from speech segments in multiple languages [57].

ITU-T G.160 Standard Voice Data: Standardized speech material associated with ITU-T Recommendation G.160 for testing voice-enhancement devices and related speech-processing systems [42].

Kayser et al. (2009) Impulse Response Database: Eight-channel database of head-related impulse responses and binaural room impulse responses measured using behind-the-ear hearing aids and an in-ear microphone on a head-and-torso simulator, including recordings from anechoic and real-world environments [40].

LibriMix Dataset: Open-source speech-separation dataset derived from clean LibriSpeech speech combined with WHAM! noise to generate noisy speech mixtures [57,60].

LibriSpeech ASR Corpus: Approximately 1000 h corpus of read English speech derived from public-domain audiobooks from LibriVox [3,23,28,39,46,50,59,60].

LRS3-TED Dataset: Large audiovisual speech corpus consisting of video clips extracted from TED and TEDx talks. It contains approximately 438 h of video data and 151,819 clips [58].

Mandarin Audio–Visual (AV) Corpus (Hou et al.): Mandarin audiovisual speech corpus based on Mandarin Hearing in Noise Test-style sentence materials, containing synchronized speech audio and facial video [30].

MUSAN Corpus: Audio corpus containing speech, music, and noise recordings, commonly used as an augmentation source for speech-processing and speaker-recognition systems [30].

NAL Speech and Noise CDs (Keidser et al., 2002): Set of three CDs produced by the National Acoustic Laboratories containing speech, background-noise, and speech-test materials [54].

National Health and Nutrition Examination Survey (NHANES) Audiograms Dataset: Audiometric data collected as part of the National Health and Nutrition Examination Survey, including pure-tone hearing thresholds used to characterize hearing status in the U.S. population [47].

NOISEX-92 Database: Classic database of recorded noise signals developed for studying the effects of additive noise on automatic speech-recognition systems. It contains 15 commonly used noise types, including babble, factory, military, vehicle, and other environmental noises [19,25,30,37,43,50,55,59,61].

Northwestern University Auditory Test No. 6 (NU-6): Standardized clinical word-recognition test consisting of lists of phonetically balanced monosyllabic words, commonly used to assess speech-recognition ability [26].

Oldenburg BTE HRIR/BRIR Database: Same underlying impulse-response database described by Kayser et al. (2009), containing multichannel in-ear and behind-the-ear HRIRs and BRIRs measured using hearing aid microphones and a head-and-torso simulator [22].

Oldenburg Sentence Test (OLSA): German matrix sentence test consisting of standardized five-word sentences used to measure speech reception thresholds in noise [3,21,57].

OLNOISE Database: Speech-shaped masking noise associated with the Oldenburg Sentence Test [21].

Slakh2100 Dataset: Synthetic multitrack music dataset containing 2100 automatically mixed tracks with aligned MIDI data. The audio is synthesized from MIDI using sample-based virtual instruments and totals approximately 145 h of mixture data [51].

Sound Ideas Sound Effects Library: Commercial sound-effects library containing professionally recorded environmental and other sound-effect recordings. In the studies in this review, it was used as a source of nonspeech environmental noise [19,37,50].

Soundsnap Sound Library: Commercial online library of sound effects and other audio recordings used as a source of environmental and background sounds [3].

Speech Test Video Corpus (STeVi): Commercial audiovisual speech corpus containing synchronized speech audio and video from English speakers, with speech materials designed for intelligibility testing [35].

Stationary Dataset (Mixkit Traffic Sound Effects): Collection of sound-effect recordings obtained from Mixkit, including traffic and other environmental or everyday sound effects [53].

Surrey Binaural Room Impulse Response (BRIR) Database: Collection of measured binaural room impulse responses recorded in real rooms at different source positions [60].

TCD-TIMIT Dataset: Large audiovisual speech corpus containing high-quality audio and video recordings of 62 speakers reading 6913 phonetically rich sentences, with video recorded from multiple viewing angles [30].

The Alchemyst Audiobook: Audiobook recording of *The Alchemyst: The Secrets of the Immortal Nicholas Flamel* used as competing speech or masker material in speech-in-noise experiments [35].

TIMIT Acoustic-Phonetic Continuous Speech Corpus: DARPA TIMIT corpus containing approximately five hours of American English read speech from 630 speakers representing eight major U.S. dialect regions, with time-aligned orthographic, phonetic, and word transcriptions [18,22,23,25,28,34,41,46,48,49,55,60].

TopCoder SpokenLanguages2 Dataset: Speech collection associated with the TopCoder SpokenLanguages2 competition, containing short speech recordings representing many different languages [59].

TSP Speech Corpus: McGill University speech database containing more than 1400 utterances from 24 speakers, with approximately equal numbers of male and female speakers. The recordings were made in an anechoic room [26,55].

UrbanSound Dataset: Urban environmental sound dataset and taxonomy developed for urban sound research, providing labeled examples of common urban sound categories [59].

UrbanSound8K Dataset: Dataset containing 8732 labeled urban sound excerpts of up to four seconds across 10 classes, including air conditioner, car horn, children playing, dog bark, drilling, engine idling, gunshot, jackhammer, siren, and street music [41,43].

USTC 115 Noises Corpus (15 Home-Made Noise Subset): Collection of 15 home noises from the larger corpus of environmental noise recordings associated with Xu et al.’s USTC speech enhancement work [43,59].

Valentini-Botinhao Speech Enhancement Dataset: Dataset that contains parallel pairs of clean and artificially corrupted speech recordings. It is composed of clean speech from the CSTR VCTK Corpus mixed with various environmental noises at specific SNRs [3].

VCTK-DEMAND Dataset: VoiceBank/VCTK speech mixed with DEMAND environmental noises; it contains 11,572 training utterances and 824 test utterances [45].

Voice Bank Corpus: Multispeaker English speech corpus that contains 28 primary English speakers plus 56 additional speakers from Scotland and the United States [43,59].

VoxCeleb2 Dataset: Large-scale audiovisual speaker-recognition dataset containing more than one million utterances from 6112 celebrities extracted from YouTube videos [3].

WHAM! Dataset: WSJ0 Hipster Ambient Mixtures dataset, consisting of WSJ0-2mix speech mixtures combined with real ambient noise recordings collected in environments such as cafés, restaurants, and bars [3,60].

WSJ0 (Wall Street Journal) Corpus: Large-vocabulary read-speech corpus based on Wall Street Journal news text [21,22,31,50].

WSJ0-SI84 Subsection Corpus: Speaker-independent training subset of the WSJ0 corpus containing approximately 7138 utterances from 83 speakers [51,61].
