# Supplementary material for: Hearing Smarter: A Scoping Review of Artificial Intelligence Implementation in Hearing Devices
Source: J Clin Med. 2026 Oct 20;15(20):8124. doi: 10.3390/jcm15208124 (PMC13705835; doi:10.3390/jcm15208124)
Supplement: Supplementary file 1 [file jcm-15-08124-s001.zip › Table S2 Electronic Database Search Strategy.pdf]

Table S2: Electronic Database Search Strategy

PubMed

Search	Query	Results
#5	#4 Filters: English, from 2010-2025	263
#4	#3 AND "Noise"[Mesh] OR "Speech Perception"[Mesh] OR "noise reduction"[tiab] OR "noise suppression"[tiab] OR "speech in noise"[tiab] OR "speech-in-noise"[tiab] OR "speech enhancement"[tiab] OR "environment* recognition"[tiab] OR "scene classification"[tiab] OR "environment classification"[tiab] OR "acoustic scene*"[tiab]	341
#3	#1 AND #2	1,621
#2	"Artificial Intelligence"[Mesh] OR "artificial intelligence"[tiab] OR "AI"[tiab] OR "A.I."[tiab] OR "machine learning"[tiab] OR "neural network*"[tiab] OR "deep learning"[tiab] OR "Bayesian"[tiab] OR "reinforcement learning"[tiab] OR "preference learning"[tiab]	585,202
#1	("Hearing Aids"[Mesh] OR "hearing aid*"[tiab] OR "hearing device*"[tiab] OR "hearing instrument*"[tiab] OR "receiver in canal"[tiab] OR "receiver-in-canal"[tiab] OR "receiver-in-the-ear"[tiab] OR "behind the ear"[tiab] "behind-the-ear"[tiab]) OR "RIC"[tiab] OR "RITE"[tiab] OR "BTE"[tiab] OR "hearing"[tiab]	141,587

Embase

Search	Query	Results
#6	#5 Filters: English, from 2010-2025	115
#5	#4 NOT 'clinical trial':dtype	129
#4	#3 AND ('noise reduction' OR 'noise suppression' OR 'speech perception' OR 'speech in noise' OR 'speech-in-noise' OR 'speech enhancement' OR 'environment* recognition' OR 'scene classification' OR 'environment* classification' OR 'acoustic scene')	135
#3	#1 AND #2	422
#2	'artificial intelligence' OR 'machine learning' OR 'deep learning' OR 'ai' OR 'a.i.' OR 'neural network*' OR 'bayesian' OR 'reinforcement learning' OR 'preference learning'	939,148
#1	'hearing aid*' OR 'hearing device*' OR 'hearing instrument' OR 'receiver in canal' OR 'receiver-in-canal' OR 'receiver in ear' OR 'receiver-in-ear' OR 'behind the ear' OR 'behind-the-ear' OR (('ric' OR 'rite' OR 'bte') AND ('hearing'/exp OR 'hearing'))	23,184

CINAHL Complete

Search	Query	Results
S5	S4 Filters: English, from 2010-2025	38

S4	S3 AND (MH "Noise" OR MH "Speech Perception") OR TI ("noise" OR "noise reduction" OR "noise suppression" OR "speech perception" OR "speech in noise" OR "speech-in-noise" OR "speech enhancement" OR "environment* recognition" OR "scene classification" OR "environment* classification" OR "acoustic scene*") OR AB ("noise" OR "noise reduction" OR "noise suppression" OR "speech perception" OR "speech in noise" OR "speech-in-noise" OR "speech enhancement" OR "environment* recognition" OR "scene classification" OR "environment* classification" OR "acoustic scene*")	43
S3	S1 AND S2	147
S2	(MH "Artificial Intelligence" OR MH "Machine Learning" OR MH "Deep Learning") OR TI ("artificial intelligence" OR "AI" OR "A.I." OR "machine learning" OR "neural network*" OR "deep learning" OR "Bayesian" OR "reinforcement learning" OR "preference learning") OR AB ("artificial intelligence" OR "AI" OR "A.I." OR "machine learning" OR "neural network*" OR "deep learning" OR "Bayesian" OR "reinforcement learning" OR "preference learning")	73,63
S1	(MH "Hearing Aids" OR MH "Hearing Aid Care") OR TI ("Hearing aid*" OR "hearing device*" OR "hearing instrument*" OR "receiver in canal" OR "receiver-in-canal" OR "reciever-in-ear" OR "behind-the-ear" OR (("RIC" OR "RITE" OR "BTE") AND ("hearing"))) OR AB ("Hearing aid*" OR "hearing device*" OR "hearing instrument*" OR "receiver in canal" OR "receiver-in-canal" OR "reciever-in-ear" OR "behind-the-ear" OR (("RIC" OR "RITE" OR "BTE") AND ("hearing")))	13,484

Web of Science

Search	Query	Results
#5	#4 Filters: English, from 2010-2025	109
#4	#3 AND ("noise reduction" OR "noise suppression" OR "speech perception" OR "speech in noise" OR "speech-in-noise" OR "speech enhancement" OR "environment* recognition" OR "scene classification" OR "environment* classification" OR "acoustic scene*")	119
#3	#1 AND #2	294
#2	"artificial intelligence" OR "machine learning" OR "deep learning" OR "AI" OR "A.I." OR "neural network*" OR "bayesian" OR "reinforcement learning" OR "preference learning"	1,332,967
#1	"hearing aid*" OR "hearing device*" OR "hearing instrument" OR "receiver in canal" OR "receiver-in-canal" OR "receiver in ear" OR "receiver-in-ear" OR "behind the ear" OR "behind-the-ear" OR (("RIC" OR "RITE” OR "BTE") AND ("hearing"))	11,971

APA PsycInfo

Search	Query	Results
S5	S4 Filters: English, from 2010-2025	26
S4	S3 AND TI ("noise" OR "noise reduction" OR "noise suppression" OR "speech perception" OR "speech in noise" OR "speech-in-noise" OR "speech enhancement" OR "environment* recognition" OR "scene classification" OR "environment* classification" OR "acoustic scene*") OR AB ("noise" OR "noise reduction" OR "noise suppression" OR "speech perception" OR "speech in noise" OR "speech-in-noise" OR "speech enhancement" OR "environment* recognition" OR "scene classification" OR "environment* classification" OR "acoustic scene*")	31
S3	S1 AND S2	48
S2	TI ("artificial intelligence" OR "AI" OR "A.I." OR "machine learning" OR "neural network*" OR "deep learning" OR "Bayesian" OR "reinforcement learning" OR "preference learning") OR AB ("artificial intelligence" OR "AI" OR "A.I." OR "machine learning" OR "neural network*" OR "deep learning" OR "Bayesian" OR "reinforcement learning" OR "preference learning")	70,409
S1	TI ("Hearing aid*" OR "hearing device*" OR "hearing instrument*" OR "receiver in canal" OR "receiver-in-canal" OR "reciever-in-ear" OR "behind-the-ear" OR (("RIC" OR "RITE" OR "BTE") AND ("hearing"))) OR AB ("Hearing aid*" OR "hearing device*" OR "hearing instrument*" OR "receiver in canal" OR "receiver-in-canal" OR "reciever-in-ear" OR "behind-the-ear" OR (("RIC" OR "RITE" OR "BTE") AND ("hearing"))	3,731
